# When healthcare becomes the barrier to heart transplantation and advanced therapies

**DOI:** 10.3389/fcvm.2025.1663498

**Published:** 2025-12-08

**Authors:** Bartlomiej R. Imielski, Aaron S. Gilani, Zohaib R. Khawaja, Barbara A. Pisani

**Affiliations:** 1 Department of Cardiothoracic Surgery, Wake Forest University School of Medicine, Winston-Salem, NC, United States; 2 Department of Cardiology, Wake Forest University School of Medicine, Winston-Salem, NC, United States

**Keywords:** heart transplantation, health insurance, UNOS, Medicare, Medicaid

## Abstract

Surgical management of advanced heart failure is complex and multi-faceted. It requires a coordinated care team and is accompanied by strict criteria for which patients qualify for either a donor heart for transplantation or a ventricular assist device. For these patients, these interventions offer the potential to extend life by multiple years. However, for patients without appropriate financial coverage, these therapies can become virtually inaccessible. Our perspective piece illustrates a real patient's struggles and highlights their complex barriers to heart transplantation.

## Introduction

Gabriella (alias for HIPAA protection) grew up defining the American dream. Having proven herself to be a caring daughter and accomplished student, she was just beginning her journey into adulthood. However, while Gabriella's motivation grew, her physical stamina began to decline. Shortly thereafter, she was diagnosed with advanced heart failure of irreversible etiology, which profoundly changed her life.

It is estimated that 6.5 million Americans over the age of 20 have heart failure (1), a clinical syndrome in which heart function is insufficient to meet the metabolic needs of the body. Heart failure is the end point for several different disease states, most commonly coronary artery disease (2), carrying a five-year survival rate of 55%. Although medical management of heart failure has significantly improved over time, it is ultimately a fatal process with a select number of patients qualifying for advanced therapies—namely heart transplantation or a ventricular assist device (VAD), a permanent implantable centrifugal impeller pump to supplement the body's cardiac output (3).

Surgical management of advanced heart failure is nuanced, requires a coordinated care team, and is accompanied by strict criteria for which patients qualify for either a donor heart or a VAD. For appropriate patients, these surgeries offer the potential to extend life by multiple years. Therefore, it became our goal to evaluate Gabriella for a heart transplant. Recipient age? Appropriate. Tobacco, alcohol, and drug usage? Nonexistent. Disqualifying comorbidities?

None. Social support? Excellent. Financial clearance? We have a problem. Unfortunately, this was not surprising given the median income of $38,000 in the United States and an average heart transplantation price tag of $750,000 (4).

Gabriella's status began to worsen, requiring multiple vasopressor agents, confined and tethered to her ICU bed by the sheer weight of her intravenous lines, indwelling catheters, and the weight of her failing body. She was an excellent candidate, but like 21 million Americans over age 18 without health insurance (5), Gabriella did not have health coverage. As a child of undocumented immigrants, she rarely considered her lack of citizenship an obstacle for someone with a trailblazer mentality. Only recently did she learn of the new challenges that accompany U.S. residents without citizenship status, but now, it would carry a life-changing designation. Suddenly, her entire care team who was keeping her alive and promising a solution were at a loss regarding how to get her approved for a heart transplant. Advanced therapies for heart failure require tremendous resources from workup to initiation of treatment, let alone longitudinal follow-up, surveillance, and subsequent management. As such, this process carries with it substantial financial considerations for patients, families, hospitals, and insurance companies who navigate these costs.

It is estimated that of the 91.4% of Americans who are insured, 68.4% have private insurance, 18.2% have Medicare, 21.1% have Medicaid, 4.9% have military coverage, and 8.6% remain uninsured (6). Hence, for a large portion of the population, insurance approval is not a rate- limiting step. However, for disadvantaged populations—and those more likely to have heart disease—healthcare coverage can become an insurmountable barrier to life-sustaining treatment, especially heart transplantation and advanced technologies.

For patients who have private insurance, either through employer-based or individual- payer plans, coverage for advanced therapies is usually nonexistent. For this cohort, financial difficulties may arise when the patient's heart failure is protracted, in which case they may lose employment and fall into coverage gaps. Another mechanism for obtaining health insurance is the Affordable Care Act (ACA). This tax-subsidized program offers individuals, regardless of working status, the ability to apply during open enrollment periods for insurance plans. However, the limitation of open enrollment or qualifying events—such as loss of a job within 60 days— opens the possibility for patients to miss their opportunity to enroll. As such, the patient is left without the ability to enroll in health insurance until the fall, when open enrollment begins and coverage commences in the new calendar year, which leads to patients either forgoing medical care or contributing to the $745 billion in unreimbursed care since 2000 (7).

Medicare offers health insurance coverage for individuals 65 years or older without private or ACA plans. For individuals younger than 65 without insurance, Medicare access is only possible if they qualify for Medicare Disability. Although this can be a routine process for patients with ESRD on dialysis or after transplant, for heart failure patients this involves a lengthy determination process, which—when approved—requires two full years of disability payments before Medicare eligibility. Once on Medicare Disability, the patient can then enroll in the ACA during open enrollment. Unfortunately, for many heart failure patients, this timeline is excessively burdensome.

Medicaid, a joint federal-state program for low-income individuals, offers another vehicle for coverage. It is income-based and, similarly to Medicare, requires disability determination, although less stringent and time-restrictive. One major challenge is balancing limited income and assets against qualifying minimums while managing ongoing hospital expenditures. It is common for a family to have sufficient income to exceed Medicaid cutoffs yet insufficient resources to meet the cost of living (8).

Lastly, military personnel qualify for Veterans Affairs (VA) benefits. However, this population may pose challenges. One recent patient who ultimately underwent LVAD implantation had never sought VA services; despite being 100% service-connected, there were delays in obtaining approval due to lack of records. Alternatively, acutely ill inpatients may receive VA approval for VADs, but outpatient candidates may require transfer to VA facilities, displacing them from home for weeks or months. This further complicates postoperative follow-up and continuity of care.

Gabriella's condition continued to worsen, requiring initiation of veno-arterial extracorporeal membrane oxygenation (VA-ECMO) life support. She qualified for Status 1 listing—critically ill on mechanical support. It was discovered that being listed as a dependent on her family's prior tax return allowed her to qualify for an individual policy during the next open enrollment period; however, this was months away. Her team pursued extraordinary measures: she was approved for institutional coverage, paving the way for future listing.

Soon after, Gabriella died on our waitlist. No suitable donor became available in time to save her life. Her family, our staff, and our community lost the promise of her future life and impact. Despite intense effort, her transplant listing was delayed due to difficulties obtaining healthcare coverage.

As healthcare providers, we must advocate for our patients in all aspects of their care— including access. Management of end-stage disease does not always result in cure, but often improves quality or prolongs life. Though we may accept the disease process as the arbiter of outcome, allowing external limitations to dictate patient fate is an insufferable reality.

Given the socioeconomic factors affecting our population—from a culture of smoking in the former tobacco belt to unemployment and poverty—a proactive approach is imperative to reduce the development of heart failure and equip patients with resources for advanced care. Our institution has developed a Community Health Worker Outreach Program, which seeks out patients who may need future transplantation or VADs and assists them in obtaining insurance if they are uninsured.

Beginning in 1973, the ESRD Medicare entitlement allowed patients younger than 65 who were receiving chronic dialysis or had undergone kidney transplant to qualify for Medicare.

Over 400,000 patients enroll annually under this stipulation (9). With heart disease being the leading cause of death in the U.S. (10), lobbying for a similar exception for heart failure patients is not unreasonable and may improve access to advanced therapies.

As of April 1, 2023, Medicaid's continuous enrollment protections implemented during the COVID-19 PHE expired, subjecting members to eligibility redetermination. It is estimated that up to 17 million Americans may lose coverage. We already care for patients on our waitlist who may be affected.

Given the complexity of our healthcare landscape, understanding access and coverage is daunting—but necessary. Not every patient is a transplant candidate, but every patient deserves to be evaluated for one.

## Data Availability

The original contributions presented in the study are included in the article/Supplementary Material, further inquiries can be directed to the corresponding author.
